# A low-cost bedside nomogram integrating TSF and pain for predicting severe malnutrition in nasopharyngeal carcinoma: a prospective cohort study

**DOI:** 10.3389/fnut.2026.1769746

**Published:** 2026-03-19

**Authors:** Shasha Shen, Kai Zhou, Guoping He, Hui Xu, Jinzhi Wu, Xin Tian, Bi Feng, Yan Lou, Hu Ma, Xiaoxia Gou

**Affiliations:** 1 Department of Head and Neck Oncology, The Second Affiliated Hospital of Zunyi Medical University, Zunyi, Guizhou, China; 2 Department of Abdominal Oncology, The Second Affiliated Hospital of Zunyi Medical University, Zunyi, Guizhou, China; 3 Department of Thoracic Oncology, The Second Affiliated Hospital of Zunyi Medical University, Zunyi, Guizhou, China

**Keywords:** INSCOC, nasopharyngeal carcinoma, nomogram, pain, severe malnutrition, Triceps Skinfold Thickness

## Abstract

**Objective:**

To examine the relationship between nutritional status and quality of life (QoL) in hospitalized patients with nasopharyngeal carcinoma (NPC), identify independent predictors of severe malnutrition, and establish a practical “low-cost” predictive nomogram to guide early clinical intervention.

**Methods:**

This study is a secondary analysis of a prospective cohort from the Investigation on Nutrition Status and Clinical Outcome of Common Cancers (INSCOC) project. We included 216 NPC patients prospectively enrolled at a participating center between January 2014 and December 2019. Clinical data, anthropometrics (including Triceps Skinfold Thickness, TSF), and patient-reported outcomes (NRS 2002 and pain scores) were collected upon admission. Patients were randomly split into a training cohort (*n* = 152) and an internal validation cohort (*n* = 64) at a 7:3 ratio. Nutritional status was assessed using the Patient-Generated Subjective Global Assessment (PG-SGA), with severe malnutrition defined as a PG-SGA score ≥ 9. Logistic regression was performed to develop the nomogram.

**Results:**

Among the 216 patients (77.8% male; mean age 50.5 ± 10.6 years), 26.9% were at nutritional risk (NRS 2002 ≥ 3), and 23.2% suffered from severe malnutrition. Worsening nutritional status was significantly associated with lower functional domain scores and higher symptom burden in QoL (*p* < 0.05). Multivariate logistic regression identified NRS 2002 score (OR = 28.52, *p* < 0.001), Pain score (OR = 2.81, *p* = 0.014), and TSF (OR = 0.83, *p* = 0.004) as independent predictors. The nomogram demonstrated strong discrimination, with an AUC of 0.903 in the training set and 0.825 in the internal validation set. Calibration curves and decision curve analysis (DCA) confirmed the model’s clinical utility.

**Conclusion:**

Based on the prospective INSCOC dataset, we developed and validated a novel, low-cost bedside nomogram integrating NRS 2002, TSF, and Pain scores. By bypassing the need for blood tests, this tool enables immediate risk stratification for NPC patients, particularly in resource-limited settings, facilitating timely and personalized nutritional interventions.

## Graphical abstract

**Figure:**
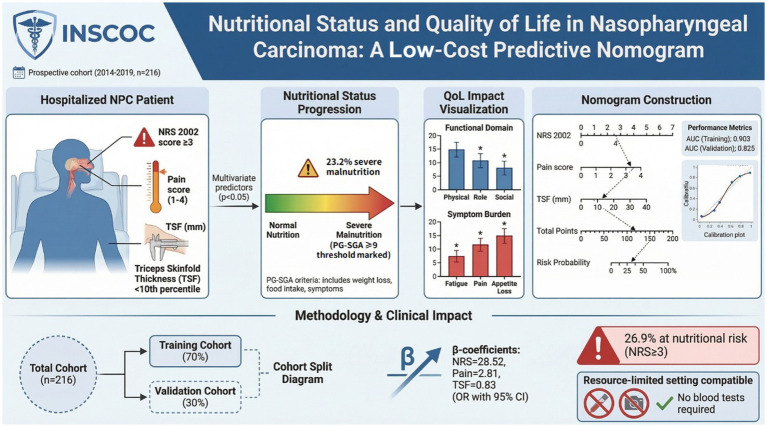

## Introduction

Nasopharyngeal carcinoma (NPC) is a distinct epithelial malignancy with a unique geographical distribution, being particularly endemic in Southeast Asia and Southern China (1). Radiotherapy, typically combined with chemotherapy, remains the cornerstone of curative treatment (2). However, the intense metabolic demands of the tumor, coupled with severe treatment-related toxicities—such as mucositis, xerostomia, and odynophagia—drastically impair oral intake, As a result, malnutrition is alarmingly prevalent in this population, with incidence rates reaching as high as 91.2% post-treatment according to Global Leadership Initiative on Malnutrition (GLIM criteria) (3). This nutritional deterioration is not merely a side effect but a critical prognostic factor, significantly associated with reduced treatment tolerance, compromised quality of life (QoL), and poor survival outcomes (4–6).

Given these severe implications, early and accurate identification of high-risk patients is paramount. Clinically, the Patient-Generated Subjective Global Assessment (PG-SGA) is widely regarded as the gold standard for nutritional assessment (7, 8). Yet, its complexity and time-consuming nature limit its routine application in high-volume oncology centers (9). Similarly, while blood-based markers (e.g., albumin, prealbumin) are objective, they are invasive, entail costs, and require laboratory turnaround time. Furthermore, these visceral proteins are heavily influenced by systemic inflammation rather than nutritional status alone, making them unreliable markers in the acute clinical setting (10, 11). In resource-limited settings or busy outpatient clinics, reliance on blood results can delay immediate clinical decision-making. Therefore, there is an urgent unmet need for a “low-cost,” non-invasive bedside tool that allows clinicians to stratify malnutrition risk instantly upon admission (12).

We hypothesize that combining basic screening (Nutritional Risk Screening 2002, NRS 2002) with simple anthropometrics (Triceps Skinfold Thickness, TSF) and patient-reported outcomes (Pain scores) can offer a rapid and effective alternative to laboratory-dependent models. TSF provides a direct estimate of fat reserves without the confounding effects of fluid retention often seen in Body Mass Index (BMI) (13, 14). Additionally, pain—an often underestimated yet common symptom in NPC—significantly contributes to reduced food intake, primarily due to the discomfort caused by mucositis (15, 16).

In this study, we utilized data from the Investigation on Nutrition Status and Clinical Outcome of Common Cancers (INSCOC) project, a large-scale prospective study in China (17, 18). By conducting a secondary analysis of a prospective cohort of NPC patients enrolled at a participating center, we aimed to: (1) examine the relationship between nutritional status and QoL; (2) identify independent determinants of severe malnutrition; and (3) develop and validate a practical nomogram based on TSF and Pain. This tool is designed to facilitate immediate, personalized nutritional intervention without reliance on laboratory facilities, thereby improving clinical accessibility and patient care efficiency.

## Materials and methods

### Study design and participants

This study was a prospective observational study conducted as part of the INSCOC project (Clinical trial registration number: ChiCTR1800020329). Patients were recruited from the Department of Head and Neck Oncology at The Second Affiliated Hospital of Zunyi Medical University (Guizhou, China) from January 1, 2014, to December 2019. As a participating center of the INSCOC project, the hospital prospectively collected clinical and nutritional data following standardized protocols (19).

The inclusion criteria were as follows: (1) age ≥ 18 years; (2) pathologically confirmed NPC; (3) conscious with the ability to communicate effectively and complete the assessments; (4) no prior nutritional intervention before admission; and (5) complete medical records and follow-up data. Patients were excluded if they had: (1) pregnancy; (2) end-stage disease with an estimated survival of < 3 months; (3) acquired immunodeficiency syndrome (AIDS); (4) admission to the intensive care unit (ICU) at the time of enrollment; or (5) a history of organ transplantation.

The study was conducted in accordance with the Declaration of Helsinki and was approved by the Ethics Committee of The Second Affiliated Hospital of Zunyi Medical University (Approval No. KYLL-2024-022). Informed consent was obtained from all participants upon enrollment in the INSCOC cohort.

### Data collection

Demographic and clinical data were collected prospectively by trained investigators within 24 h of admission. Collected variables included sociodemographic characteristics (age, sex, ethnicity, marital status, education level, smoking, alcohol and tea consumption, residence, and insurance type) and clinical characteristics (tumor stage, comorbidities, treatment modality, family history, and length of hospital stay).

Anthropometric measurements were performed by trained nurses using standardized techniques to minimize inter-observer variability. Body weight and height were measured to calculate BMI. TSF was measured using skinfold calipers at the midpoint between the acromion and olecranon on the non-dominant arm. To ensure data reliability, inter-observer consistency was assessed prior to the study, yielding an Intraclass Correlation Coefficient (ICC) of 0.92. Each TSF measurement was performed three times, and the mean value was used for analysis. Mid-arm circumference (MAC) and handgrip strength (HGS) were also recorded (20–22).

Nutritional Risk and Status Assessment were mandated by the INSCOC protocol. Nutritional risk was screened within 24 h of admission using the NRS-2002, with a score ≥3 indicating nutritional risk as defined in international guidelines (12, 23). Nutritional status was further evaluated within 48 h using the PG-SGA, a validated cancer-specific nutritional assessment tool. In PG-SGA, patients are categorized as well-nourished (0–1), suspected malnutrition (2–3), moderate malnutrition (4–8), or severe malnutrition (≥9) based on the cumulative score, with severe malnutrition (PG-SGA ≥ 9) as the primary outcome in this study (24). In this study, NRS 2002 and PG-SGA were utilized to represent distinct stages of the clinical nutritional care pathway. NRS 2002 was employed as a rapid, mandatory first-step screening tool to identify patients at nutritional risk upon admission. In contrast, the PG-SGA was used as the diagnostic gold standard to determine the severity of malnutrition. By incorporating NRS 2002 into the predictive nomogram, we aimed to bridge the clinical gap between initial screening and comprehensive diagnostic confirmation, thereby providing a weighted tool for prioritizing patients for intensive intervention.

### Quality of life assessment

QoL was evaluated using the European Organization for Research and Treatment of Cancer Quality of Life Questionnaire Core 30 (EORTC QLQ-C30) Version 3.0. For the correlation analysis between nutritional status and QoL, scores were linearly transformed to a 0–100 scale according to the EORTC scoring manual (25, 26). Notably, for the development of the bedside nomogram, we utilized the raw score of Item 9 (“Have you had pain?”) rather than the transformed score. This item is rated on a 4-point Likert scale (1 = Not at all, 2 = A little, 3 = Quite a bit, 4 = Very much). Using the raw score eliminates the need for complex calculation, ensuring the model remains a “low-cost” and immediate screening tool (27, 28).

### Statistical analysis

All statistical analyses were performed using SPSS version 29.0 (IBM Corp., Armonk, NY, USA) and R software version 4.2.3 (29). Continuous variables were expressed as mean ± standard deviation (SD) or median with interquartile range (IQR), and categorical variables as frequencies and percentages. Differences between groups were analyzed using ANOVA, the Kruskal–Wallis test, or the Chi-square test, as appropriate. Pearson correlation analysis was used to assess the relationship between nutritional status and QoL domains (30).

To develop the predictive model, the total cohort was randomly split into a training set and an internal validation set at a ratio of 7:3 using a computer-generated random number sequence (R software version 4.2.3). The sample size for this study was determined by the available data from the prospective INSCOC project during the study period. A post-hoc power analysis indicated that with 59 outcome events and 3 final predictors, the events per variable (EPV) ratio was 19.7 for the total cohort and 14.7 for the training set (*n* = 152). This meets the minimum requirement for developing stable logistic regression models. Furthermore, according to the criteria proposed by Riley et al. (31), a sample size of approximately 150 patients is sufficient to achieve a global shrinkage factor of ≥ 0.9 and minimize overfitting, given the observed event rate and number of predictors. Univariate logistic regression was performed to identify potential risk factors for severe malnutrition (32). Variables with a *p*-value < 0.05 were included in the multivariate logistic regression model. Before constructing the final model, multicollinearity among the candidate independent variables was assessed using the Variance Inflation Factor (VIF); a threshold of VIF < 5 was considered to indicate no severe multicollinearity. Specifically, all variables included in the final model demonstrated VIF values below 2.0, confirming that despite the strong predictive weight of NRS 2002, there was no significant multicollinearity, thus ensuring the statistical stability of the multivariate model (33).

Based on the multivariate analysis, a nomogram was constructed (34). The discriminative ability of the model was assessed using the area under the receiver operating characteristic (ROC) curve (AUC). To further account for potential overfitting and provide a more robust assessment of the model’s performance, internal validation was enhanced using bootstrapping with 1,000 resamples. This technique was used to calculate the optimism-corrected Area Under the Curve (AUC) and the calibration slope, ensuring the model’s stability despite the modest number of outcome events (35). Calibration was evaluated using calibration plots and the Hosmer–Lemeshow test. Decision curve analysis (DCA) was performed to determine the clinical utility of the nomogram (36). A two-sided *p*-value < 0.05 was considered statistically significant.

## Results

### Demographic and clinical characteristics

A total of 216 patients with NPC were included in the final analysis. The cohort was predominantly male (77.78%) with a mean age of 50.5 ± 10.6 years. Most patients (87.96%) were under 65 years of age. Regarding nutritional status, 26.85% (*n* = 58) of patients were identified as being at nutritional risk (NRS 2002 ≥ 3) upon admission. However, despite this risk, the rate of nutritional intervention was critically low; only 13.79% (8/58) of the at-risk patients received nutritional support during hospitalization (Table 1), highlighting a significant gap between risk identification and clinical management.

**Table 1:** Baseline characteristics of hospitalized NPC patients (*n* = 216).

Variable	*n* (%)
Gender	
Male	168 (77.78)
Female	48 (22.22)
Age	
<65 years	190 (87.96)
≥65 years	26 (12.04)
Marital status	
Unmarried	6 (2.78)
Married	206 (95.37)
Divorced/Widowed	4 (1.85)
Ethnicity	
Han	190 (87.96)
Others	26 (12.03)
Family history of cancer	
Yes	17 (7.87)
No	199 (92.13)
Place of residence	
Urban/Town	98 (45.37)
Rural	118 (54.63)
Clinical stage	
Stage I	4 (1.85)
Stage II	13 (6.02)
Stage III	77 (35.65)
Stage IV	122 (56.48)
Education level	
No schooling/Primary School	113 (52.31)
Middle/High School	97 (44.91)
College or above	6 (2.78)
Smoking history	
Yes	117 (54.17)
No	99 (45.83)
Alcohol consumption	
Yes	44 (20.37)
No	172 (79.63)
Tea consumption	
Yes	70 (32.41)
No	146 (67.59)
Comorbidities	
Yes	49 (22.69)
No	167 (77.31)
Nutritional support	
Parenteral nutrition	10 (4.63)
Enteral nutrition	1 (0.47)
None	205 (94.90)
Treatment modality	
Chemotherapy	104 (48.15)
Radiotherapy	17 (7.87)
Chemoradiotherapy	49 (22.68)
Chemoradio + Targeted/Immuno	6 (2.78)
None	40 (18.52)
Length of hospital stay	
<12 days	106 (49.07)
≥12 days	110 (50.93)
NRS 2002 score	
<3 points	158 (73.15)
≥3 points	58 (26.85)

### Nutritional risk stratification and support status

**Figure 1:**
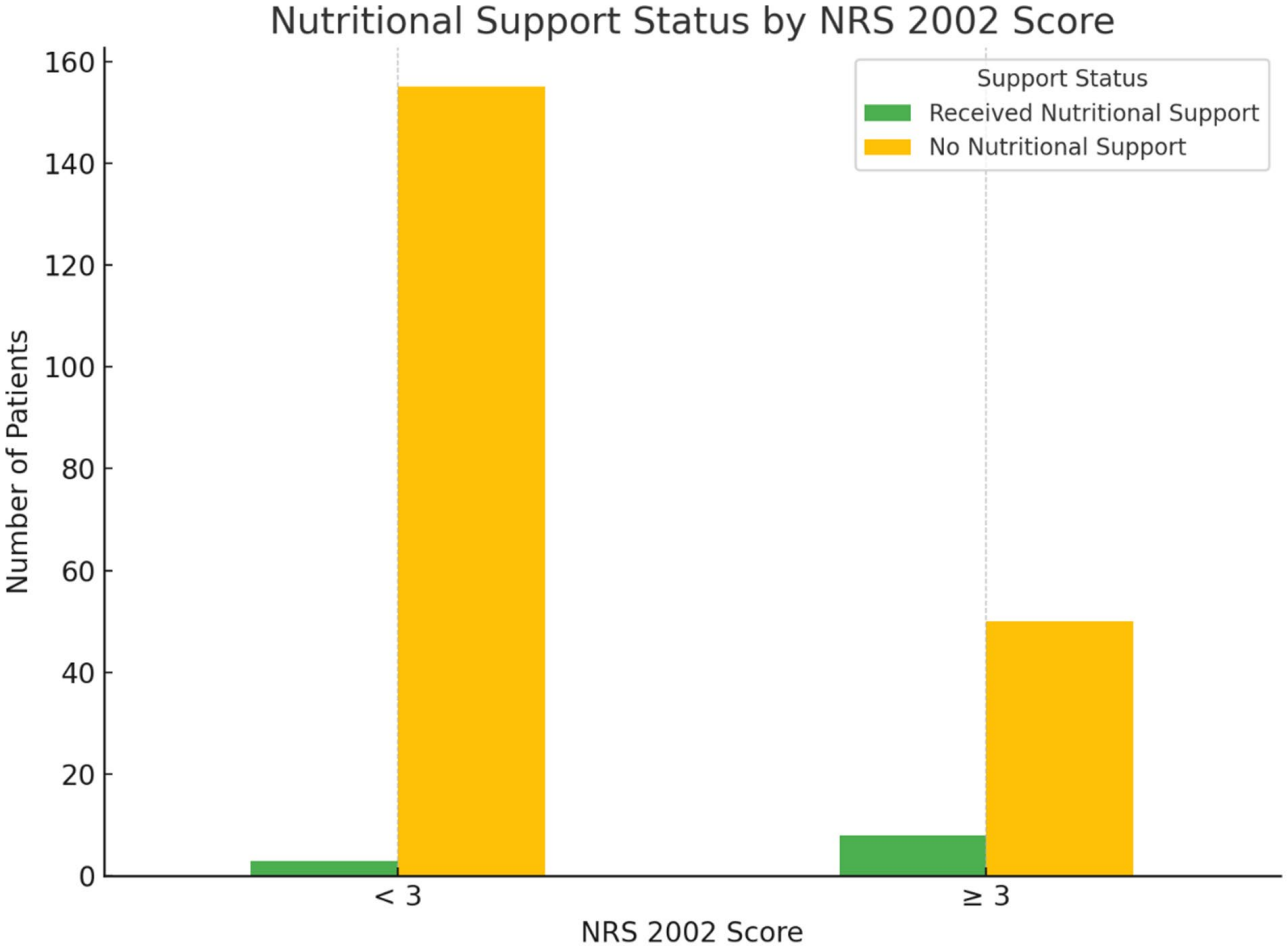
Nutritional support status stratified by NRS 2002 risk categories. Green bars represent patients who received nutritional support (enteral or parenteral), and yellow bars represent patients who did not receive nutritional support. Patients were grouped as non-risk (NRS 2002 < 3) or at-risk (NRS 2002 ≥ 3).

Among the 216 patients, 158 (73.15%) were classified as having no nutritional risk (NRS 2002 < 3), and 58 (26.85%) were at nutritional risk (NRS 2002 ≥ 3). Despite recommendations, only 8 patients (13.79%) in the at-risk group received nutritional support. A chi-square test revealed a significant association between risk classification and support implementation (χ^2^ = 10.08, *p* = 0.001); however, the absolute intervention rate remained critically low (Figure 1).

### Comparison of physical measurements and biochemical indicators

As PG-SGA scores increased (indicating worsening nutritional status), physical measurements including weight, BMI, MAC, TSF, and ND-HGS decreased significantly (all *p* < 0.05). Biochemical analysis revealed that Prealbumin, PAR, and NLR exhibited significant variations across different nutritional categories (*p* < 0.05). In contrast, no statistically significant differences were observed in Red Blood Cell (RBC) count (*p* = 0.052) or Blood Urea Nitrogen (BUN) levels (*p* = 0.087) relative to nutritional status, although a downward trend in RBC count was noted as malnutrition progressed (Tables 2, 3).

**Table 2:** Comparison of physical measurements among patients with different PG-SGA scores.

Variable	PG-SGA			*F*	*p*-value
	0–3 (*n* = 104)	4–8 (*n* = 62)	≥9 (*n* = 50)		
Weight (kg)	61.23 ± 10.52	58.78 ± 8.25	55.94 ± 9.40	5.191	0.006
Body Mass Index (kg/m^2^)	23.06 ± 3.19	22.63 ± 2.83	21.33 ± 3.20	5.328	0.006
Mid-Arm Circumference (MAC, cm)	25.96 ± 3.28	25.21 ± 2.64	24.42 ± 2.79	4.609	0.011
Triceps Skinfold Thickness (TSF, mm)	11.78 ± 8.63	10.65 ± 7.00	8.12 ± 6.83	3.711	0.026
Non-Dominant Hand Grip Strength (ND-HGS, kg)	27.03 ± 9.63	25.94 ± 9.13	21.98 ± 8.95	5.022	0.007
Mid-Arm Muscle Circumference (MAMC, cm)	22.26 ± 3.30	21.87 ± 3.32	21.87 ± 3.52	0.369	0.692
Max Calf Circumference (Right, cm)	34.32 ± 4.39	33.27 ± 2.36	31.75 ± 3.60	8.061	<0.001
Max Calf Circumference (Left, cm)	34.09 ± 3.18	33.18 ± 2.30	31.60 ± 3.54	11.251	<0.001

**Table 3:** Comparison of biochemical indicators among patients with different PG-SGA scores.

Variable	PG-SGA			*F*	*p*-value
	0–3 (*n* = 104)	4–8 (*n* = 62)	≥9 (*n* = 50)		
Albumin (g/L)	39.63 ± 4.13	38.91 ± 4.60	38.33 ± 4.88	1.555	0.214
Prealbumin (mg/L)	234.46 ± 63.38	219.48 ± 74.16	195.98 ± 58.50	5.84	0.003
Hemoglobin (g/L)	127.05 ± 18.72	125.11 ± 19.85	120.64 ± 18.46	1.925	0.148
White blood cell count (×10^9^/L)	5.41 ± 2.02	5.37 ± 2.06	6.15 ± 3.38	1.856	0.159
Neutrophil count (×10^9^/L)	3.19 ± 1.51	3.26 ± 1.65	4.25 ± 3.08	5.024	0.007
Red blood cell count (×10^12^/L)	4.28 ± 0.61	4.10 ± 0.72	4.04 ± 0.61	3.004	0.052
Creatinine (μmol/L)	77.61 ± 23.22	77.95 ± 31.24	76.80 ± 17.80	0.031	0.969
Blood urea nitrogen (mmol/L)	4.87 ± 2.11	5.86 ± 2.95	5.43 ± 3.41	2.476	0.087
Neutrophil-to-WBC ratio (NLR)	0.58 ± 0.12	0.60 ± 0.14	0.64 ± 0.14	3.500	0.032
Total-to-direct bilirubin ratio (TDR)	4.11 ± 2.85	3.96 ± 1.14	4.64 ± 4.02	0.876	0.416
Prealbumin-to-albumin ratio (PAR)	5.88 ± 1.32	5.58 ± 1.68	5.08 ± 1.40	5.168	0.006

### Comparison of QoL among patients with different nutritional statuses

A comparison of EORTC QLQ-C30 scores across different PG-SGA nutritional status groups revealed that functional scores declined progressively with worsening nutritional status. Patients with PG-SGA scores ≥9 exhibited significantly lower scores in physical, role, emotional, cognitive, and social functioning (all *p* < 0.01). Notably, global health status was also substantially compromised in this group (*Z* = 51.936, *p* < 0.001). Simultaneously, symptom scores increased with higher PG-SGA levels. Fatigue, pain, nausea/vomiting, insomnia, appetite loss, and constipation were all significantly more prevalent among patients with moderate to severe malnutrition (PG-SGA ≥ 4) (*p* < 0.01 for all). No significant variation was observed across groups in dyspnea, diarrhea, or financial burden (*p* > 0.05). These findings confirm that nutritional deterioration in NPC patients is strongly associated with both impaired functioning and higher symptom burden, underscoring the clinical importance of early nutritional risk identification and intervention (Table 4).

**Table 4:** EORTC QLQ-C30 functional and symptom domains by PG-SGA categories.

Domain	PG-SGA			Z	*p*-value
	0–3 (*n* = 104)	4–8 (*n* = 62)	≥9 (*n* = 50)		
Functional domains					
Physical functioning	93.33 (86.67, 100.00)	90.00 (80.00, 100.00)	73.33 (60.00, 86.67)	44.491	<0.001
Role functioning	100.00 (66.67, 100.00)	75.00 (66.67, 100.00)	66.67 (50.00, 66.67)	34.528	<0.001
Emotional functioning	91.67 (75.00, 100.00)	83.33 (75.00, 100.00)	75.00 (66.67, 91.67)	14.897	0.001
Cognitive functioning	83.33 (83.33, 100.00)	83.33 (66.67, 100.00)	66.67 (66.67, 87.50)	10.711	0.005
Social functioning	66.67 (66.67, 79.17)	66.67 (66.67, 66.67)	66.67 (33.33, 66.67)	30.886	<0.001
Global health status/QoL	66.67 (66.67, 83.33)	66.67 (50.00, 66.67)	50.00 (33.33, 50.00)	51.936	<0.001
Symptom domains					
Fatigue	11.11 (11.11, 22.22)	22.22 (11.11, 33.33)	33.33 (22.22, 44.44)	42.921	<0.001
Nausea and vomiting	0.00 (0.00, 0.00)	0.00 (0.00, 16.67)	16.67 (0.00, 33.33)	17.43	<0.001
Pain	0.00 (0.00, 16.67)	16.67 (0.00, 33.33)	33.33 (0.00, 33.33)	18.258	<0.001
Dyspnea	0.00 (0.00, 0.00)	0.00 (0.00, 0.00)	0.00 (0.00, 33.33)	2.975	0.226
Insomnia	0.00 (0.00, 33.33)	0.00 (0.00, 33.33)	33.33 (0.00, 66.67)	12.05	0.002
Appetite loss	0.00 (0.00, 0.00)	33.33 (0.00, 33.33)	33.33 (0.00, 66.67)	50.39	<0.001
Constipation	0.00 (0.00, 0.00)	0.00 (0.00, 33.33)	33.33 (0.00, 33.33)	20.04	<0.001
Diarrhea	0.00 (0.00, 0.00)	0.00 (0.00, 0.00)	0.00 (0.00, 0.00)	2.266	0.322
Financial difficulties	33.33 (33.33, 66.67)	33.33 (33.33, 66.67)	33.33 (33.33, 66.67)	2.83	0.243

### Correlation analysis between nutritional status and QoL

**Figure 2:**
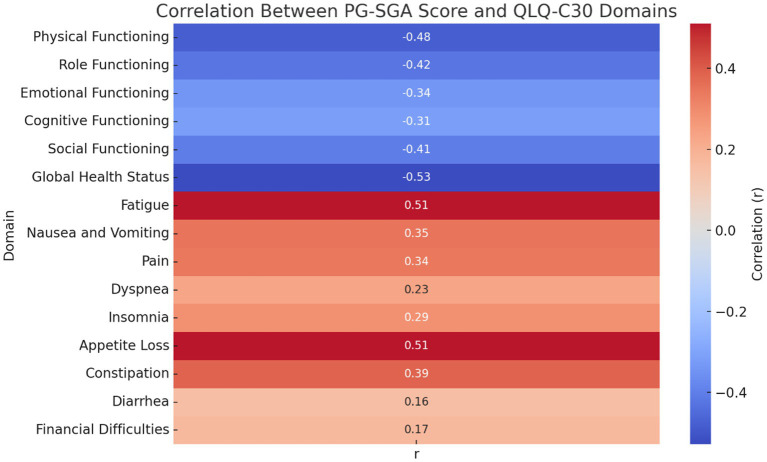
Correlation between PG-SGA score and EORTC QLQ-C30 domains. The color gradient reflects the strength and direction of the Pearson correlation coefficient (*r*) between PG-SGA scores and QLQ-C30 domains. Red indicates a positive correlation (worsening symptoms), while blue indicates a negative correlation (declining function). *p*-values < 0.05 were considered statistically significant.

Correlation analysis showed that the PG-SGA score was negatively associated with all functional domains of the QLQ-C30, including physical (*r* = −0.480), role (*r* = −0.424), emotional (*r* = −0.339), cognitive (*r* = −0.313), and social functioning (*r* = −0.405), as well as overall QoL (*r* = −0.528), all with *p* < 0.001. This indicates that worse nutritional status is strongly correlated with lower QoL and functional capacity. Conversely, the PG-SGA score was positively correlated with symptom burden, particularly fatigue (*r* = 0.509), appetite loss (*r* = 0.511), and constipation (*r* = 0.389), among others. Significant associations were also observed for nausea and vomiting, pain, dyspnea, and insomnia (all *p* < 0.05). Diarrhea and financial difficulties showed weaker but still statistically significant correlations (*r* = 0.156, *p* = 0.021; *r* = 0.172, *p* = 0.011, respectively) (Figure 2).

### Development of a predictive model for severe malnutrition

#### Flowchart of model construction

**Figure 3:**
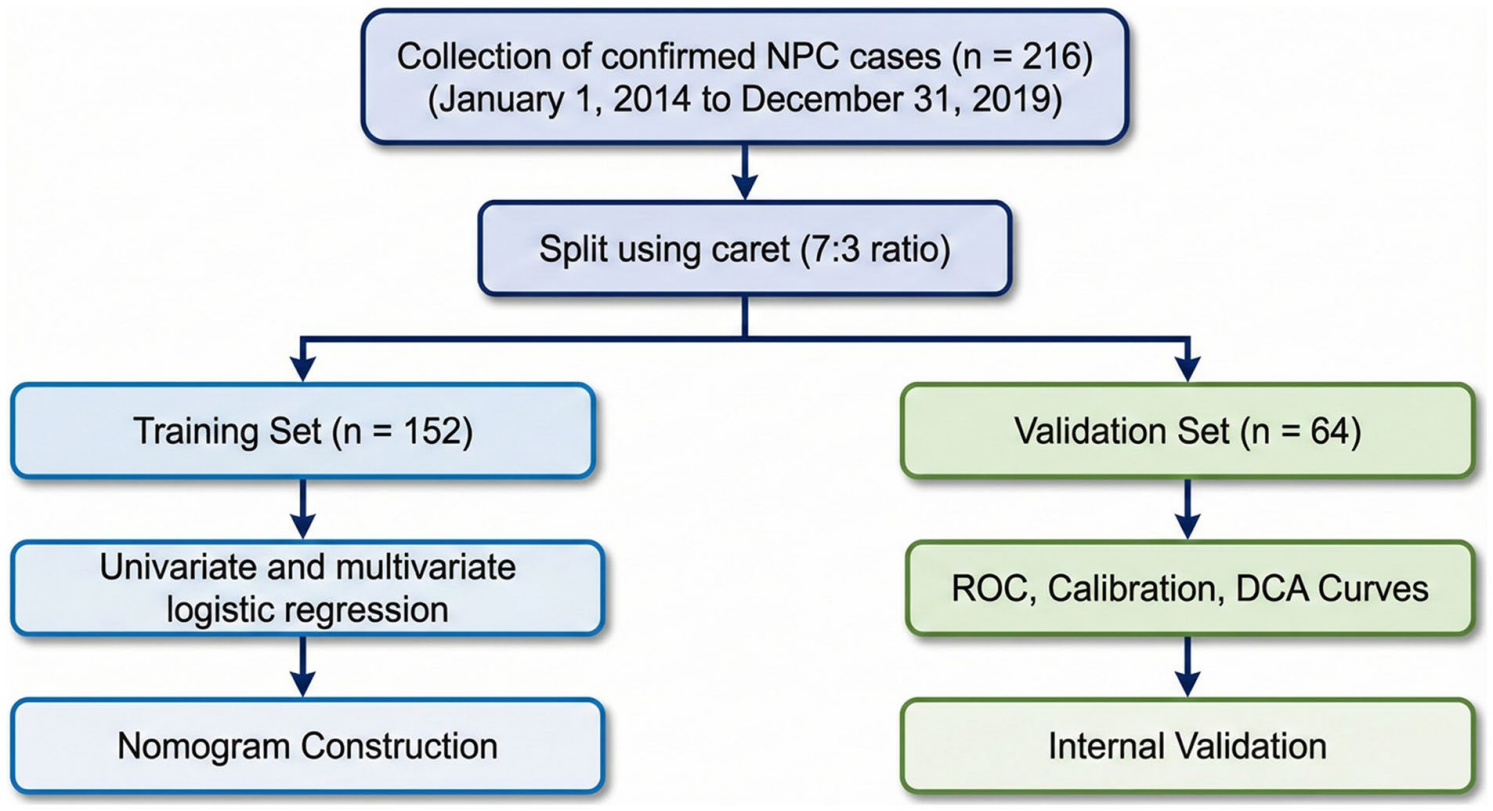
Technical roadmap of predictive model for severe malnutrition. The diagram outlines the patient selection process, the randomization of the cohort into training (*n* = 152) and validation (*n* = 64) sets, and the statistical workflow used to develop and validate the predictive nomogram.

The study workflow is illustrated in Figure 3. A total of 216 eligible patients were included in the final analysis. Based on randomization, 152 patients were assigned to the training cohort to develop the nomogram, and 64 patients were assigned to the validation cohort for internal validation.

### Univariate analysis of factors associated with severe malnutrition

**Figure 4:**
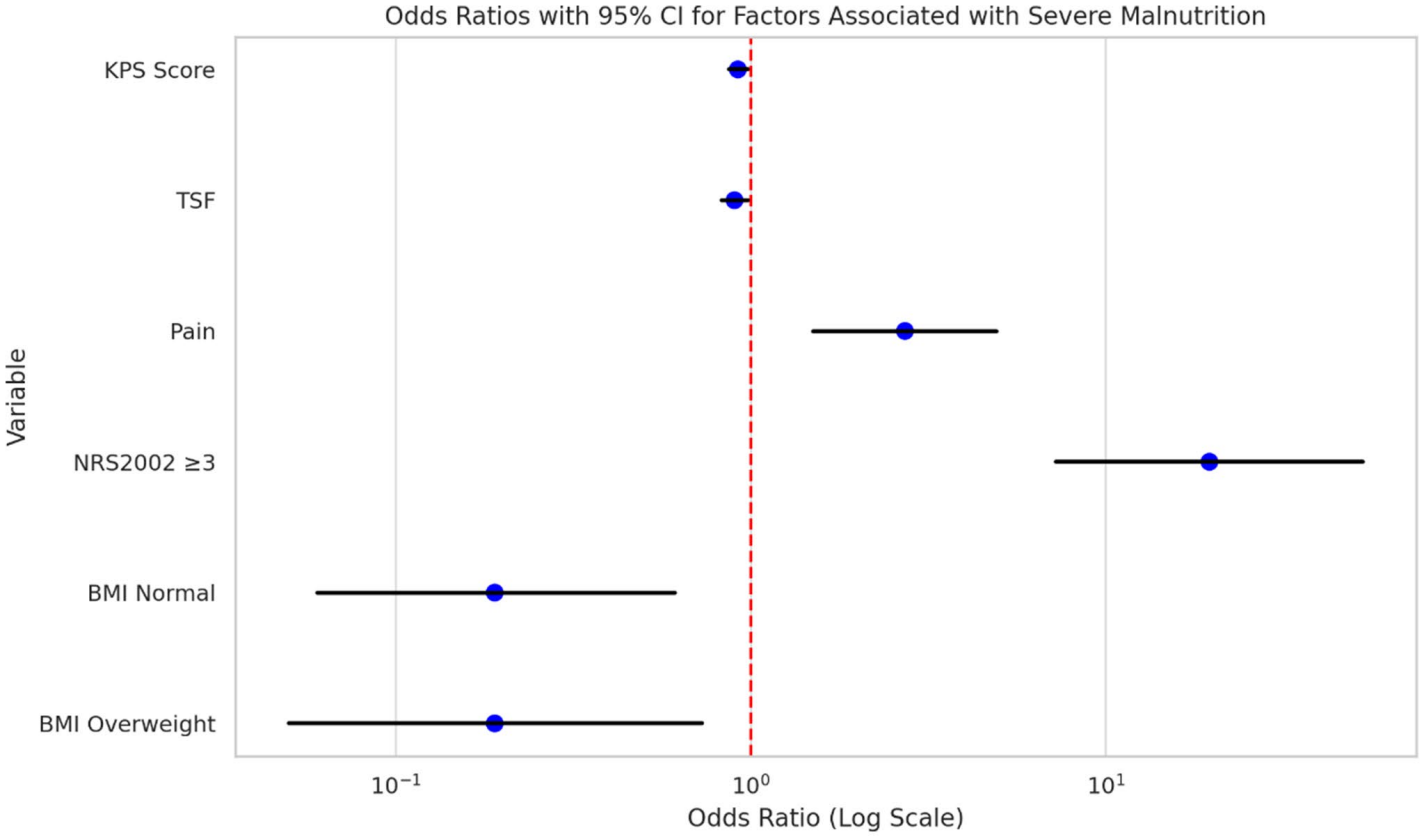
Risk factors for severe malnutrition in NPC patients. The plot displays the odds ratios (OR) and 95% confidence intervals (CI) for risk factors associated with severe malnutrition. Variables with *p* < 0.05 (e.g., KPS, TSF, Pain, NRS 2002) were considered potential predictors.

Univariate logistic regression analysis was performed to identify factors associated with severe malnutrition among hospitalized NPC patients (Table 5; Figure 4). The results showed that a lower Karnofsky Performance Status (KPS) score (OR = 0.92, 95% CI: 0.87–0.98, *p* = 0.012), lower TSF (OR = 0.90, 95% CI: 0.83–0.98, *p* = 0.010), presence of pain (OR = 2.72, 95% CI: 1.50–4.93, *p* = 0.001), and NRS2002 score ≥3 (OR = 19.60, 95% CI: 7.25–52.94, *p* < 0.001) were significantly associated with increased odds of severe malnutrition. In contrast, patients with normal BMI (OR = 0.19, 95% CI: 0.06–0.61, *p* = 0.006) and those classified as overweight (OR = 0.19, 95% CI: 0.05–0.73, *p* = 0.015) were less likely to develop severe malnutrition. In the univariate analysis, factors such as gender, age, comorbidities, and clinical stage were not found to be significantly associated with severe malnutrition (all *p* > 0.05).

**Table 5:** Univariate logistic regression analysis of factors influencing severe malnutrition.

Variable	*β*	S. E.	OR	95% CI	*Z*	*p*-value
Gender	0.347	0.537	1.41	0.49–4.05	0.647	0.518
Age	0.792	0.544	2.21	0.76–6.41	1.455	0.146
Comorbidities	−0.212	0.504	0.81	0.30–2.17	−0.421	0.674
Family history of cancer	0.161	0.687	1.17	0.31–4.52	0.235	0.815
Smoking history	0.234	0.418	1.26	0.56–2.87	0.561	0.575
KPS score	−0.081	0.032	0.92	0.87–0.98	−2.517	0.012
Total protein	−0.001	0.034	1	0.93–1.07	−0.035	0.972
Albumin	−0.072	0.047	0.93	0.85–1.02	−1.545	0.122
MAC	−0.132	0.076	0.88	0.75–1.02	−1.742	0.081
TSF	−0.103	0.04	0.9	0.83–0.98	−2.578	0.01
MAMC	0.022	0.064	1.02	0.90–1.16	0.348	0.728
Pain	1	0.304	2.72	1.50–4.93	3.293	0.001
NRS2002 score	2.975	0.507	19.6	7.25–52.94	5.874	<0.001
Total bilirubin	−0.002	0.05	1	0.91–1.10	−0.03	0.976
BMI category						
Normal BMI	−1.674	0.604	0.19	0.06–0.61	−2.771	0.006
Overweight	−1.638	0.676	0.19	0.05–0.73	−2.424	0.015

#### Multivariate logistic regression analysis of risk factors for severe malnutrition

**Figure 5:**
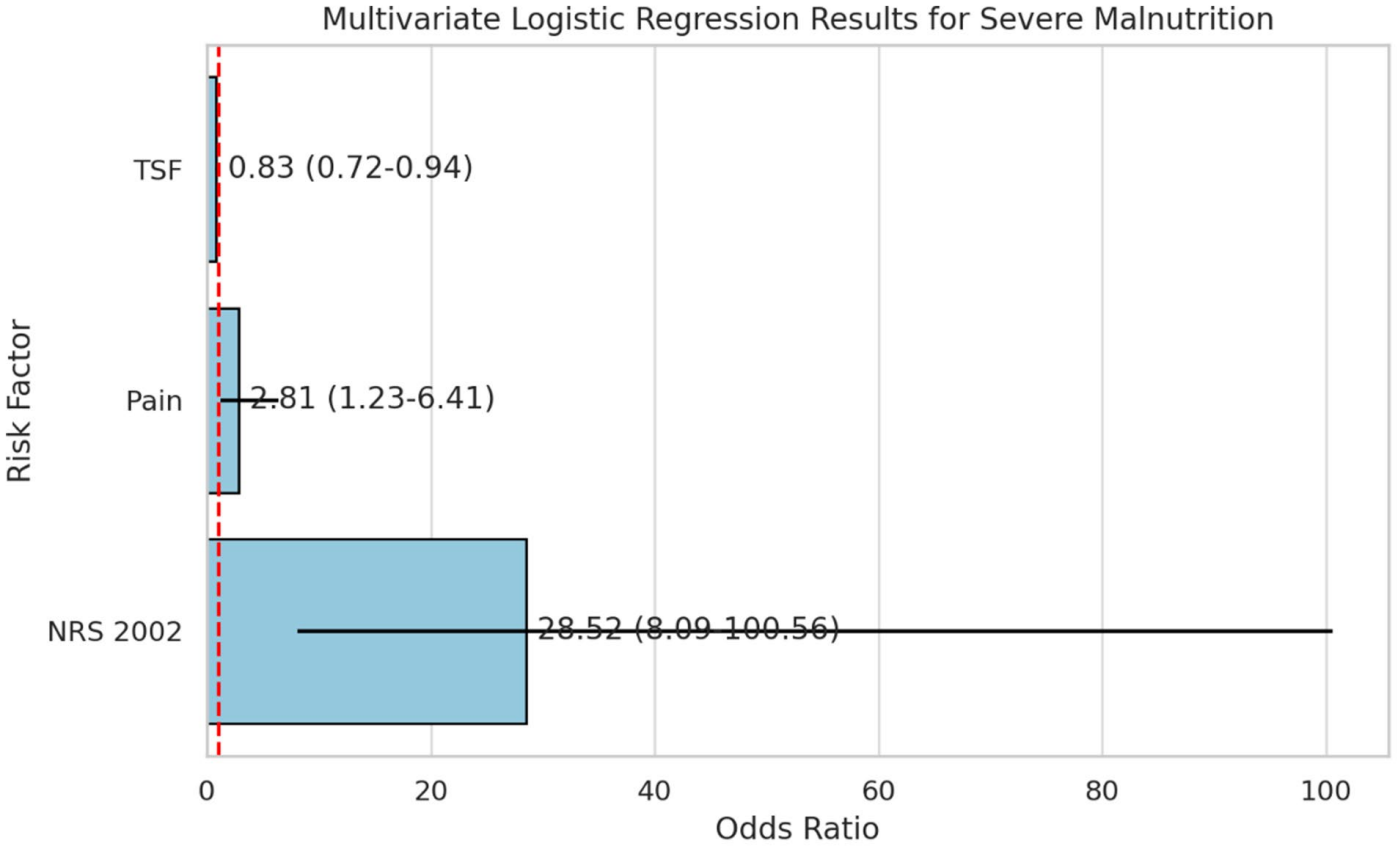
Forest plot of independent risk factors for severe malnutrition. This plot identifies the independent predictors of severe malnutrition included in the final model. Pain score (OR = 2.81), NRS 2002 score (OR = 28.52), and TSF (OR = 0.83) were statistically significant. The horizontal lines represent the 95% CI.

Multivariate logistic regression was conducted to identify independent risk factors for severe malnutrition among hospitalized NPC patients. Variables with *p* < 0.05 in the univariate analysis—including KPS score, pain, TSF, BMI, and NRS-2002 score—were entered into the model. Collinearity diagnosis revealed that the VIF values for these variables were all within the acceptable range (< 5), ruling out significant multicollinearity. The results demonstrated that higher pain scores (OR = 2.81, 95% CI: 1.23–6.41, *p* = 0.014) and higher NRS-2002 scores (OR = 28.52, 95% CI: 8.09–100.56, *p* < 0.001) were significantly associated with an increased risk of severe malnutrition. Conversely, greater TSF was a protective factor (OR = 0.83, 95% CI: 0.72–0.94, *p* = 0.004) (Table 6; Figure 5). Although KPS score and BMI were significant in the univariate analysis, they were not identified as independent predictors in the multivariate model.

**Table 6:** Multivariate logistic regression for predictors of severe malnutrition in NPC patients.

Variable	*β*	S. E.	OR	95% CI	*Z*	*p*-value
TSF	−0.192	0.067	0.83	0.72–0.94	−2.872	0.004
Pain	1.032	0.421	2.81	1.23–6.41	2.454	0.014
NRS-2002	3.35	0.643	28.52	8.09–100.56	5.213	<0.001

#### Construction of the clinical nomogram

**Figure 6:**
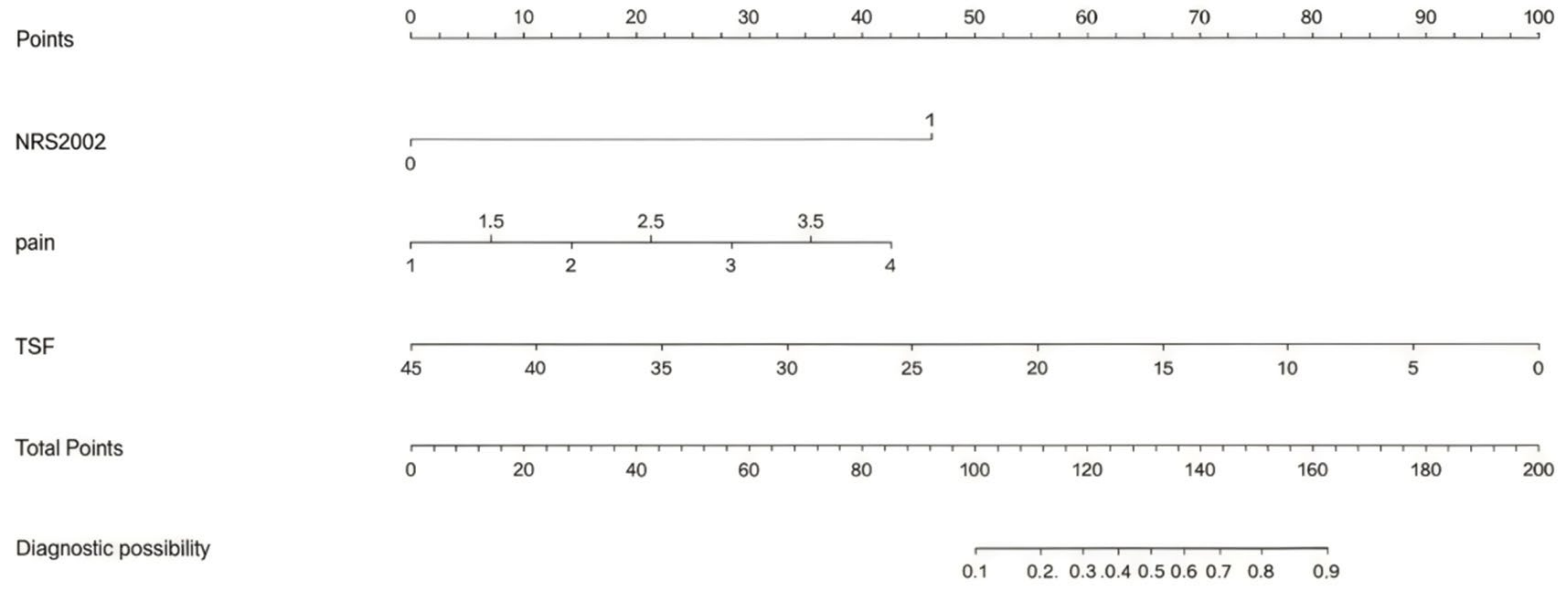
Nomogram for predicting severe malnutrition in patients with NPC. To use the nomogram: (1) Locate the patient’s value for each variable (NRS 2002, pain, TSF) on its respective axis. (2) Draw a vertical line upward to the “Points” axis to determine the score for each variable. (3) Sum the points and locate the total on the “Total Points” axis. (4) Draw a vertical line downward to the “Diagnostic possibility” axis to obtain the estimated probability of severe malnutrition. Worked Example: a patient with TSF of 10 mm (35 pts), pain score of 2 (20 pts), and NRS 2002 of 3 (80 pts) results in a total score of 135, corresponding to a > 70% predicted probability of severe malnutrition.

Based on the results of multivariate logistic regression analysis, we developed a predictive model for severe malnutrition incorporating three independent variables: pain score, TSF, and NRS 2002 score. This model allowed for individualized risk prediction of severe malnutrition in hospitalized NPC patients. To facilitate its clinical application, we subsequently constructed a predictive nomogram, as shown in Figure 6.

### Evaluation of the nomogram’s clinical performance

**Figure 7:**
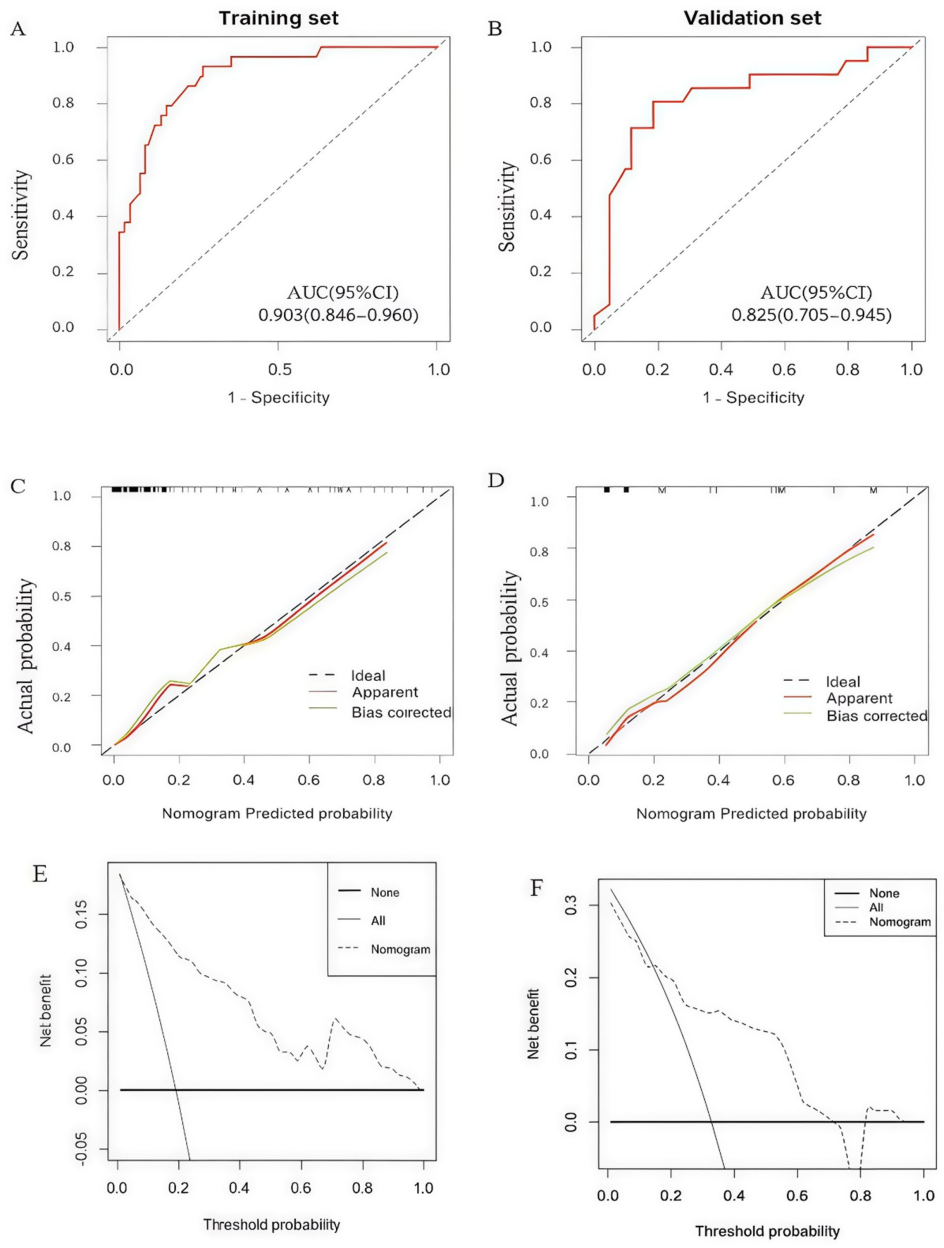
Model performance and clinical utility evaluation: **(A)** ROC curve for the training cohort; **(B)** ROC curve for the internal validation cohort; **(C)** calibration plot for the training cohort; **(D)** calibration plot for the internal validation cohort; **(E)** decision curve analysis (DCA) for the training cohort; **(F)** DCA for the internal validation cohort. AUC, Area under the curve.

The discriminative ability of the nomogram was evaluated using ROC curves. In the training cohort, the model demonstrated excellent performance with an AUC of 0.903 (95% CI: 0.846–0.960) (Figure 7A). After 1,000 bootstrap iterations to account for potential overfitting, the optimism-corrected AUC was found to be 0.895. Consistent with these findings, the internal validation cohort also exhibited robust discrimination, yielding an AUC of 0.825 (95% CI: 0.705–0.945) (Figure 7B). To further evaluate the independent predictive value of the novel bedside markers, a sensitivity analysis was conducted by excluding NRS 2002. The reduced model, incorporating only Pain, TSF, and KPS, still maintained meaningful discriminative ability with an AUC of 0.701. This demonstrates that while NRS 2002 is a dominant predictor, the integrated bedside variables provide significant, non-redundant information for risk stratification.

Calibration was assessed using calibration plots and the Hosmer–Lemeshow test. The bootstrap-estimated mean calibration slope was 1.121, indicating strong model stability. The calibration curves for both the training (Figure 7C) and validation cohorts (Figure 7D) showed high agreement between the predicted probabilities and actual observations, with the bias-corrected lines tracking closely to the ideal 45-degree line. The Hosmer–Lemeshow test yielded a non-significant *p*-value of 0.862, confirming the model’s good fit.

Furthermore, DCA was performed to evaluate the clinical utility of the nomogram. In the training cohort, the DCA curve showed that the nomogram provided a greater net benefit than “treat-all” or “treat-none” strategies across a wide range of threshold probabilities (> 0.03) (Figure 7E). Similarly, in the internal validation cohort, the DCA confirmed that using the nomogram to predict severe malnutrition yielded superior net clinical benefit, particularly within the threshold probability range of approximately 0.15 to 0.75 (Figure 7F). These results substantiate the practical value of the nomogram for clinical decision-making.

## Discussion

Malnutrition is pervasive among patients with head and neck cancers, with NPC posing a particularly high risk due to unique anatomical and treatment-related challenges and high nutrition impact symptom burden, which often impairs oral intake and weight maintenance (37). In our cohort derived from the INSCOC project, we observed that while 26.85% of patients were flagged as “at risk” by NRS-2002, a substantially greater proportion were identified as having moderate-to-severe malnutrition by PG-SGA, highlighting the critical gap between routine screening and comprehensive nutritional assessment (38). Furthermore, deteriorating nutritional status has been repeatedly linked to poorer QoL and greater symptom burden in cancer patients, consistent with findings reported by Polański et al. and others in mixed oncology populations (39–41). Similarly, our recent study on lung cancer, which used PG-SGA scores and biochemical data, confirmed that nutritional risk and functional decline are closely associated with increased symptom burden, further underscoring the significance of comprehensive nutritional screening and timely intervention (42).

A key finding of our study is the identification of pain as a potent, independent driver of severe malnutrition. In head and neck cancers, especially NPC, pain is rarely isolated; it frequently arises as a manifestation of severe radiation-induced oral mucositis or extensive tumor invasion, directly leading to odynophagia and mechanical inability to eat, thereby reducing oral intake and contributing to weight loss and malnutrition (43). Nutrition impact symptoms, including oral pain, dysphagia, and anorexia, have been shown to correlate with significantly lower energy and nutrient intake in head and neck cancer populations, reinforcing the role of symptom burden in nutritional deterioration (44). Radiotherapy-induced mucosal injury triggers a vicious cycle in which pain and swallowing difficulties limit intake, exacerbating mucosal breakdown and further intensifying pain and feeding impairment. This cycle suggests that effective pain management should not be viewed merely as palliative care but as a fundamental component of nutritional therapy and overall supportive care in this patient group (45).

To address the need for rapid risk stratification, we constructed a practical nomogram integrating Pain, TSF, and NRS-2002. Similar predictive models incorporating combinations of nutritional, inflammatory, and clinical indicators have been shown to provide reliable individualized risk prediction in cancer populations (46). We intentionally excluded laboratory-based inflammatory markers (e.g., albumin, NLR); although such markers have prognostic value, they require invasive sampling and often entail processing delays that limit their immediate utility in busy outpatient or resource-limited settings (47). By relying solely on anthropometrics (TSF) and patient-reported outcomes (Pain), our model serves as a “low-cost” bedside tool that allows clinicians to identify high-risk patients instantly upon admission. Moreover, we prioritized TSF over BMI in our final model. BMI can be confounded by tumor mass or treatment-induced fluid retention and may not accurately reflect adipose reserves, whereas TSF—as an established measure of subcutaneous fat—provides a more direct and reliable estimate of fat stores in cancer patients (48, 49). We acknowledge the conceptual overlap between the NRS 2002 screening tool and the PG-SGA assessment tool. However, in clinical practice, they serve distinct roles: NRS 2002 is a mandatory, rapid first-step screen, while PG-SGA is a diagnostic gold standard that is often too time-consuming for routine use (50, 51). By integrating NRS 2002 with TSF and Pain scores, our nomogram serves as a weighted bridge, helping clinicians prioritize which ‘at-risk’ patients require immediate, intensive nutritional intervention. The high odds ratio for NRS 2002 reflects its established sensitivity, but the inclusion of anthropometrics and patient-reported pain adds a layer of objective, clinical insight that traditional screening alone lacks (48, 52–54).

Regarding the treatment distribution in our cohort, the relatively high proportion of patients receiving chemotherapy alone (48.15%) reflects the timing of our baseline assessment at admission, often during the induction chemotherapy phase. Furthermore, our hospital is located in a developing region of Southwest China where a high burden of locally advanced disease is prevalent (55). As shown in Table 1, over 90% of our participants presented with stage III–IV disease. In such settings, induction chemotherapy followed by radiotherapy is a frequently utilized strategy to achieve early systemic control for extensive tumor loads (56, 57). By assessing patients at this baseline stage, our model captures the “pre-treatment” nutritional risk driven by advanced tumor burden and systemic inflammation, which is essential for identifying those who may not tolerate subsequent intensive concurrent chemoradiotherapy (58).

Furthermore, while malnutrition in NPC patients often reaches its nadir during or after concurrent chemoradiotherapy (CCRT), the clinical value of early prediction at admission lies in proactive risk stratification. Identifying high-risk patients at the baseline allows clinicians to implement ‘nutritional pre-habilitation’ strategies (59, 60). Specifically, for patients flagged by the nomogram, actionable interventions may include prophylactic placement of feeding tubes, early initiation of high-protein oral nutritional supplements (ONS), and more frequent multidisciplinary monitoring. Such early interventions are critical for maintaining the nutritional reserve required to tolerate the toxicities of CCRT, thereby reducing treatment interruptions and potentially improving long-term clinical outcomes (61–63).

In the context of modern clinical nutrition, our nomogram can be seamlessly integrated with the GLIM framework. While GLIM is the current gold standard for the diagnosis of malnutrition, our tool functions as an early-stage risk stratification system (64). Specifically, patients identified as high-risk by the nomogram can be prioritized for a formal GLIM assessment to confirm the diagnosis and grade the severity of malnutrition (55). This stepped-care approach ensures that intensive nutritional interventions—such as those defined by GLIM-based metabolic support—are initiated early in the treatment trajectory, thereby optimizing the use of clinical resources and improving patient tolerance to concurrent chemoradiotherapy (57, 58).

Our study has several strengths and limitations. A major strength is the use of prospectively collected data from the INSCOC project, which ensures standardized assessment protocols and high data quality compared to traditional retrospective chart reviews (65). Regarding limitations, this was a secondary analysis of a single-center cohort. However, our hospital is located in Southwest China, a recognized endemic region for NPC, with epidemiological patterns markedly different from non-endemic regions (66). Consequently, our cohort represents a homogeneous population with specific genetic and lifestyle characteristics distinct from non-endemic areas, minimizing potential confounders often seen in multi-center studies spanning diverse populations (67). While external validation is warranted in future multi-center trials, the internal validation (bootstrap/split-sample) confirms the reliability of our nomogram within this specific high-risk demographic.

Despite the robust performance metrics, several limitations must be acknowledged. First, although our post-hoc analysis suggests that the sample size (*n* = 216) and the number of outcome events (*n* = 59) were adequate for the three final predictors (EPV = 14.7 in the training set), the event rate remains modest. This may limit the ability of the model to capture more complex non-linear relationships and could still predispose the model to potential overfitting (68). Therefore, this work should be interpreted as exploratory and hypothesis-generating rather than a definitive clinical tool (69). Second, we acknowledge that the data were collected between 2014 and 2019. While NPC treatment protocols have evolved—notably with the integration of immunotherapy—the physiological drivers of malnutrition, such as pain and TSF depletion, remain consistent markers of nutritional deterioration (70). Nonetheless, further validation in a contemporary, multi-center cohort treated with modern protocols is essential to confirm the continued generalizability of our nomogram (71).

In conclusion, this prospective study identified Pain, TSF, and NRS 2002 as key predictors of severe malnutrition in NPC patients. While the resulting nomogram demonstrated high discriminative ability, its clinical application should be approached with caution given the exploratory nature of the study. These findings provide a valuable foundation for future large-scale research to refine and externally validate robust predictive tools for clinical oncology.

## Data Availability

The original contributions presented in the study are included in the article/supplementary material, further inquiries can be directed to the corresponding authors.
